# Behavioral Risk Profiles of Stroke Survivors Among US Adults: Geographic Differences Between Stroke Belt and Non–Stroke Belt States

**DOI:** 10.5888/pcd21.240113

**Published:** 2024-10-03

**Authors:** Derek Liuzzo, Nancy Fell, Gregory Heath, Preeti Raghavan, David Levine

**Affiliations:** 1 Department of Physical Therapy, College of Health, Education, and Professional Studies, University of Tennessee at Chattanooga; 2 Department of Health and Human Performance, College of Health, Education, and Professional Studies, University of Tennessee at Chattanooga; 3 Department of Internal Medicine, University of Tennessee Health Science Center College of Medicine, Chattanooga; 4 Department of Physical Medicine and Neurology, Johns Hopkins Medicine, Baltimore, Maryland

## Abstract

**Introduction:**

Stroke, a leading cause of illness, death, and long-term disability in the US, presents with significant disparities across the country, most notably in southeastern states comprising the “Stroke Belt.” This study intended to identify differences between Stroke Belt states (SBS) and non–Stroke Belt states (NSBS) in terms of prevalence of stroke, sociodemographic and behavioral risk factors, and health-related quality of life (HRQOL).

**Methods:**

We analyzed data from the 2019 Behavioral Risk Factor Surveillance System to compare demographic characteristics, risk factors, physical activity adherence, functional independence, and HRQOL among stroke survivors in SBS and NSBS.

**Results:**

Of 18,745 stroke survivors, 4,272 were from SBS and 14,473 were from NSBS. Stroke was more prevalent in SBS (odds ratio [OR] = 1.39; 95% CI, 1.35–1.44; *P* < .001), with significant differences by age, sex, and race and ethnicity, except for Hispanic ethnicity. Selected stroke risk factors were more common in every category in SBS. Stroke survivors in SBS were less likely to meet physical activity guidelines for aerobic (OR = 0.77; 95% CI, 0.69–0.86; *P* < .001) and aerobic and strengthening combined (OR = 0.77; 95% CI, 0.70–0.86; *P* < .001) activities. Stroke survivors in SBS were more likely to not meet either physical activity guideline (OR = 1.31; 95% CI, 1.22–1.41; *P* < .001).

**Conclusions:**

Living in SBS significantly increased the odds of stroke occurrence. Stroke survivors from SBS reported lower HRQOL and insufficient physical activity as well as lower functional independence. Specific strategies are needed for residents of SBS, with a focus on policies and primary and secondary prevention practices across healthcare professions.

**Summary**

**What is already known on this topic?**

Despite a decrease in stroke prevalence in the early 2000s, incidence of stroke and disability following stroke are increasing, especially in the southeastern US.

**What is added by the report?**

We analyzed 2019 Behavioral Risk Factor Surveillance System data on stroke survivors for the nation and the “Stroke Belt” and examined the odds of having and/or living with a disability from a stroke by demographic group, social determinants of health, and health-related quality of life following a stroke. Stroke was more prevalent in Stroke Belt states (SBS), and significant differences were seen by age, gender, and race and ethnicity.

**What are the implications for public health practice?**

State and local public health professionals, especially those in SBS, can allocate and prioritize funding to create educational initiatives and stroke preventive measures.

## Introduction

Stroke is a leading cause of illness, death, and long-term disability in the US (1). At the beginning of the 21st century, 63.3 (95% CI, 56.9–66.6) Americans per 100,000 population were dying from stroke, with disability-adjusted life years (DALYs) estimated to be 1,205.2 (95% CI, 1,112.2–1,287.9) per 100,000 population (2–5). A downward trend reached a nadir in 2010, with a 23.2% decrease in stroke deaths and 13.7% decrease in DALYs (1,4). However, from 2010 to 2019, a 12.4% rise in deaths (168,680 in 2019) and 10.1% increase in DALY occurred (1,4). Although mortality rates remain at 8.8% less than the peak in 2000, stroke survivors are living longer with disability and rates of DALYs continue to rise (3–5). These events pose enormous challenges to survivors and their care providers and families, as well as the health care system, with medical costs exceeding $53 billion (1).

Historically, a higher stroke incidence and prevalence is evidenced in the southeastern US, also known as “Stroke Belt” states (SBS) (5–7). One criterion for classifying a state as one of the SBS is when stroke incidence and deaths exceed 10% above the median rate for all states (5,6). Eight states have consistently met this criterion since 1999: Alabama, Arkansas, Georgia, Louisiana (except for 2000), Mississippi, North Carolina, South Carolina, and Tennessee (5–7).

This study’s purpose was to investigate the differences in self-reported stroke prevalence, sociodemographic characteristics, and modifiable cardiovascular and behavioral risk factors among US stroke survivors in SBS and NSBS by analyzing data from the 2019 Behavioral Risk Factor Surveillance System (BRFSS). Our goal is to use study results to support development of data-driven primary and secondary prevention interventions to mitigate risk and reduce stroke disparity in SBS.

## Methods

The BRFSS is a state-based surveillance system conducted by the Centers for Disease Control and Prevention (CDC) that collects data from US residents about their health-related risk behaviors, chronic health conditions, and use of preventive health services. Stroke survivor data are collected annually and can be used to help determine factors leading to an initial stroke or the recurrence of stroke in the US (5,8). A more detailed description of the survey design and random-sampling procedures is available from a review by Pierannunzi et al (9).

We defined the SBS as the 8 states identified by the REGARDS study (REasons for Geographic and Racial Differences in Stroke): Alabama, Arkansas, Georgia, Louisiana, Mississippi, North Carolina, South Carolina, and Tennessee (5).

2019 BRFSS data were downloaded directly from the CDC’s BRFSS website by using SAS Transport and imported into SPSS (IBM Corporation) for descriptive analyses. We calculated odds ratios (ORs) to illustrate the differences in stroke prevalence between stroke survivors in SBS and stroke survivors in NSBS. For the variables of demographic characteristics, social determinants of health, modifiable cardiovascular risk factors, physical activity, disability in terms of functional independence, and health-related quality of life (HRQOL), ORs were generated only for respondents who replied that they had experienced a stroke. Total respondent data included 18,745 stroke survivors, 4,272 from SBS and 14,473 from NSBS; however, total numbers for some variables did not equal the full sample size because of incomplete responses. Because stroke risk increases with the presence of specific conditions and disease processes (eg, cardiovascular, metabolic), behavioral risk factors, and social determinants of health, associations between these variables were examined to explore differences in SBS compared with NSBS. 

Sociodemographic variables analyzed included age, sex, race and ethnicity, income, education, and health care coverage. Health care coverage was assessed via the following BRFSS questions: “Do you have any kind of health care coverage, including health insurance, prepaid plans such as HMOs, or government plans such as Medicare, or Indian Health Service?” and “Was there a time in the past 12 months when you needed to see a doctor but could not because of cost?” Modifiable cardiovascular risk factor and lifestyle variables analyzed included body mass index (BMI, kg/m^2^), hypertension, type 2 diabetes, depression, cholesterol levels, and smoking (8). 

Respondents also answered BRFSS questions about their adherence to the 2018 Physical Activity Guidelines for Americans: performing 150 minutes or more per week of moderate aerobic physical activity and/or engaging in exercises to strengthen the muscles for 20 minutes at least 2 days per week (10). With reference to the guidelines, respondents were classified into 4 groups on the basis of their participation in these 2 modes of physical activity: meeting both aerobic and strengthening guidelines, meeting strengthening guidelines only, meeting aerobic guidelines only, and not meeting either guideline (10).

Daily functional abilities were determined through a series of BRFSS questions that included, “Do you have serious difficulty walking or climbing stairs?”, “Do you have difficulty dressing or bathing?”, and “Because of a physical, mental, or emotional condition, do you have difficulty doing errands alone such as visiting a doctor’s office or shopping?” (8).

Respondents were also asked the following questions related to HRQOL: “Now thinking about your mental health, which includes stress, depression, and problems with emotions, for how many days during the past 30 days was your mental health not good?”, “Have you ever been told you had a depressive disorder (including depression, major depression, dysthymia, or minor depression)?”, and “Now thinking about your physical health, which includes physical illness and injury, for how many days during the past 30 days was your physical health not good?” (11).

We used a series of stratified 2 × 2 tables to calculate Mantel-Haenszel χ^2^ statistics, using an α level of .05. For prevalence of stroke, stratification included data from all respondents: those reporting a history of stroke as well as those without stroke history. For each of the following comparisons, only respondents with a history of stroke were assessed. We calculated ORs and 95% CIs and used univariate analyses to illustrate the broad, overarching factors contributing to stroke prevalence in SBS versus NSBS.

## Results

The 2019 BRFSS complete data set included 418,268 respondents, 18,745 of whom identified as stroke survivors (4.5%) (Table 1). Comparing stroke prevalence across states, those living in SBS had significantly higher odds (OR = 1.39; 95% CI, 1.35–1.44; *P* < .001) of having a stroke (5.7%) compared with NSBS (4.2%). 

**Table 1:** Prevalence of Stroke and Non-Stroke in Stroke Belt States and Non–Stroke Belt States, by Age Group, Behavioral Risk Factor Surveillance System, 2019^a^^,^ b

Stroke status	Total, % (n)	Age group, % (n)	
		18–64 y	≥65 y
Total respondents in SBS	17.7 (73,862)	11.0 (46,023)	6.4 (26,674)
Total respondents in NSBS	82.3 (344,406)	50.8 (212,566)	29.9 (125,138)
Total respondents^b^	100 (418,268)	61.8 (258,589)	36.3 (151,812)
**Respondents with stroke^b^**			
SBS	5.7 (4,272)	3.7 (1,680)	9.6 (2,552)
NSBS	4.2 (14,473)	2.4 (5,112)	7.4 (9,199)
Total	4.4 (18,543)	2.6 (6,792)	7.7 (11,751)
**Respondents without stroke^b^**			
SBS	94.3 (69,620)	96.3 (44,343)	90.4 (24,122)
NSBS	93.6 (322,238)	97.6 (207,454)	92.6 (115,939)
Total	93.7 (391,858)	97.4 (251,797)	92.3 (140,061)
**SBS vs NSBS (reference group)**			
Mantel-Haenszel χ^2^	—	294.0	151.2
*P* value	<.001	<.001	<.001
OR (95% CI)	1.39 (1.35–1.44)	1.54 (1.45–1.63)	1.33 (1.27–1.40)

Abbreviations: NSBS, Non–Stroke Belt states; OR, odds ratio; SBS, Stroke Belt states.

a SBS are Alabama, Arkansas, Georgia, Louisiana, Mississippi, North Carolina, South Carolina, and Tennessee.

b Percentages in each section may not sum to 100% because of some incomplete responses.

### Demographic characteristics and social determinants of health

A higher proportion of respondents aged 18 to 64 years in SBS had experienced stroke compared with those in NSBS (OR = 1.54; 95% CI, 1.45–1.63; *P* < .001). Men (OR = 1.33; 95% CI, 1.26–1.41) and women (OR = 1.44; 95% CI, 1.38–1.51) in SBS were more likely to have had a stroke than men and women in NSBS (*P* < .001 for both). Non-Hispanic White, Non-Hispanic Black, and American Indian or Alaska Native respondents in SBS had significantly higher odds of having a stroke than their counterparts in NSBS (OR range, 1.21–1.92) (Table 2). However, Hispanic or Latino individuals in SBS had lower odds (OR = 0.76; 95% CI, 0.57–0.99; *P* < .05) of having a stroke. Across all education levels, respondents in SBS had significantly higher odds of experiencing a stroke than those in NSBS, and the odds decreased as education increased (OR range, 1.68–1.19). We also found significant differences between SBS and NSBS regarding annual household income; respondents in SBS in the highest (>$75,000 per year: OR = 1.39; 95% CI, 1.25–1.53) and lowest income categories (<$20,000 per year: OR = 1.42; 95% CI, 1.33–1.52) had higher odds of stroke than their counterparts in NSBS (*P* < .001 for both). Most respondents in both SBS and NSBS reported having health insurance. Respondents in SBS who reported that the medical cost of health care was a barrier for seeking treatment had higher odds of stroke than respondents in NSBS (OR = 1.38; 95% CI, 1.26–1.52; *P* < .001) (Table 2).

**Table 2:** Demographics and Social Determinants of Health for Stroke Respondents in Stroke Belt and Non–Stroke Belt States, Behavioral Risk Factor Surveillance System, 2019^a^^,^ b

Respondent characteristic	Stroke respondents (n = 18,745)	Stroke survivors in SBS (n = 4,272)	Stroke survivors in NSBS (n = 14,473)	OR (95% CI)^c^
	n (%)			
**Age, y**				
18–64	6,792 (36.6)	1,680 (39.7)	5,112 (35.7)	1.54 (1.45–1.63)^d^
≥65	11,751 (63.4)	2,552 (60.3)	9,199 (64.3)	1.33 (1.27–1.40)^d^
**Sex**				
Male	8,494 (45.3)	1,803 (42.2)	6,691 (46.2)	1.33 (1.26–1.41)^d^
Female	10,251 (54.7)	2,469 (57.8)	7,782 (53.8)	1.44 (1.38–1.51)^d^
**Race and ethnicity**				
White, Non-Hispanic	13,809 (79.6)	2,925 (72.4)	10,884 (81.8)	1.31 (1.26–1.37)^d^
Black, Non-Hispanic	2,082 (12.0)	968 (24.0)	1,114 (8.4)	1.21 (1.11–1.33)^d^
American Indian or Alaskan Native	490 (2.8)	92 (2.3)	398 (3.0)	1.92 (1.51–2.50)^d^
Hispanic or Latino	964 (5.6)	55 (1.4)	909 (6.8)	0.76 (0.57–0.99)^e^
**Education**				
Did not complete high school	2,346 (12.6)	752 (17.7)	1,594 (11.1)	1.68 (1.53–1.84)^d^
Graduated from high school	5,950 (31.9)	1,424 (33.5)	4,526 (31.4)	1.35 (1.27–1.44)^d^
Attended college or technical school	5,596 (30.0)	1,195 (28.1)	4,401 (30.6)	1.32 (1.23–1.41)^d^
Graduated from college or technical school	4,759 (25.5)	881 (20.7)	3,878 (26.9)	1.19 (1.11–1.29)^d^
**Annual household income, $**				
<20,000	4,799 (32.5)	1,252 (38.9)	3,547 (30.7)	1.42 (1.33–1.52)^d^
20,000 to <35,000	3,977 (27.0)	828 (25.7)	3,149 (27.3)	1.21 (1.12–1.31)^d^
35,000 to <50,000	1,921 (13.0)	369 (11.5)	1,552 (13.5)	1.16 (1.03–1.31)^d^
50,000 to <75,000	1,762 (11.9)	315 (9.8)	1,447 (12.5)	1.12 (0.99–1.27)^e^
>75,000	2,296 (15.6)	456 (14.2)	1,840 (16.0)	1.39 (1.25–1.53)^d^
**Health insurance**				
Yes	17,705 (94.9)	3,999 (94.3)	13,706 (95.1)	1.24 (1.07–1.44)^e^
No	949 (5.1)	243 (5.7)	706 (4.9)	
**Medical cost^f^**				
Yes	2,495 (13.4)	700 (16.4)	1,795 (12.4)	1.38 (1.26–1.52)^d^
No	16,181 (86.6)	3,557 (83.6)	12,624 (87.6)	

Abbreviations: NSBS, Non–Stroke Belt states; OR, odds ratio; SBS, Stroke Belt states.

a SBS are Alabama, Arkansas, Georgia, Louisiana, Mississippi, North Carolina, South Carolina, and Tennessee.

b Values for n may not sum to total because of nonresponse.

c ORs assess differences between SBS and NSBS.

d *P* < .001 calculated using χ^2^ test.

e *P* < .05 calculated using χ^2^ test.

f Refers to whether respondents avoided seeing a physician or seeking medical assistance because of the cost.

### Modifiable cardiovascular risk factors

Risk factors for stroke were higher in almost all categories in SBS versus NSBS (Table 3). BMI odds ratios ranged from 0.89 to 1.17 and were higher in underweight (OR = 1.17; 95% CI, 0.93–1.46; not significant) and obese respondents (OR = 1.11; 95% CI, 1.03–1.20; *P* < .01) but lower in normal weight (OR = 0.89; 95% CI, 0.82–0.96; *P* < .01) and overweight (OR = 0.98; 95% CI 0.91–1.05; not significant) respondents.

**Table 3:** Lifestyle and Modifiable Risk Factors Among Stroke Respondents in Stroke Belt and Non–Stroke Belt States, Behavioral Risk Factor Surveillance System, 2019^a^^,^ b

Respondent characteristic	Total stroke respondents (n = 18,745)	Stroke survivors in SBS (n = 4,272)	Stroke survivors in NSBS (n = 14,473)	OR (95% CI)
	n (%)			
**Body mass index, kg/m^2 ^**	**(n = 17,481) **	**(n = 3,977) **	**(n = 13,504) **	**—**
<18.5	404 (2.3)	103 (2.6)	301 (2.2)	1.17 (0.93–1.46)
18.5–24.9	4,652 (26.6)	989 (24.9)	3,663 (27.1)	0.89 (0.82–0.96)^c^
25.0–29.9	6,094 (34.9)	1,369 (34.4)	4,725 (35.0)	0.98 (0.91–1.05)
≥30.0	6,331 (36.2)	1,516 (38.1)	4,815 (35.7)	1.11 (1.03–1.20)^c^
**History of depressive disorders **	**(n = 18,586) **	**(n = 4,243) **	**(n = 14,343)**	**—**
Yes	5,553 (29.9)	1,374 (32.4)	4,179 (29.1)	1.17 (1.08–1.25)^d^
No	13,033 (70.1)	2,869 (67.6)	10,164 (70.9)	
**History of hypertension **	**(n = 18,662) **	**(n = 4,253) **	**(n = 14,409)**	**—**
Yes	13,624 (73.0)	3,322 (78.1)	10,302 (71.5)	1.42 (1.31–1.54)^d^
No	5,038 (27.0)	931 (21.9)	4,107 (28.5)	
**History of type 2 diabetes **	**(n = 17,971) **	**(n = 4,116) **	**(n = 13,855)**	**—**
Yes	6,074 (33.8)	1,557 (37.8)	4,517 (32.6)	1.26 (1.17–1.35)^d^
No	11,897 (66.2)	2,559 (62.2)	9,338 (67.4)	
**Serum cholesterol >200 mg/dL **	**(n = 17,285) **	**(n = 3,680) **	**(n = 13,335)**	**—**
Yes	10,459 (60.5)	2,251 (63.8)	7,938 (59.5)	1.20 (1.11–1.29)^d^
No	6,826 (39.5)	1,429 (36.2)	5,397 (40.5)	
**Current smoker **	**(n = 17,950) **	**(n = 4,084) **	**(n = 13,866)**	**—**
Yes	3,456 (19.3)	911 (22.3)	2,545 (18.4)	1.28 (1.17–1.39)^d^
No	14,494 (80.7)	3,173 (77.7)	11,321 (81.6)	

Abbreviations: —, not applicable; NSBS, Non–Stroke Belt states; OR, odds ratio; SBS, Stroke Belt states.

a SBS are Alabama, Arkansas, Georgia, Louisiana, Mississippi, North Carolina, South Carolina, and Tennessee.

b Values for n may not sum to total because of refused or did not know.

c ORs assess differences between SBS and NSBS; χ^2^ *P* value <.01.

d ORs assess differences between SBS and NSBS; χ^2^ *P* value < .001.

Respondents in SBS also had a higher prevalence of history of depressive disorders (OR = 1.17; 95% CI, 1.08–1.25; *P* < .001), hypertension (OR = 1.42; 95% CI, 1.31–1.54; *P* < .001), type 2 diabetes (OR = 1.26; 95% CI, 1.17–1.35; *P* < .001), serum cholesterol over 200 mg/dL (OR = 1.20; 95% CI, 1.11–1.29; *P* < .001), and being a current smoker (OR = 1.28; 95% CI, 1.17–1.39; *P* < .001) (Table 3).

### Physical activity, disability, and health-related quality of life

Compared with stroke survivors in NSBS, stroke survivors in SBS were less likely to meet the national physical activity aerobic guideline (OR = 0.77; 95% CI, 0.69–0.86; *P* < .001) or both guidelines combined (OR = 0.77; 95% CI, 0.70–0.86; *P* < .001). Stroke survivors in SBS were more likely to not meet either physical activity guideline (OR = 1.31; 95% CI, 1.22–1.41; *P* < .001) (Table 4).

**Table 4:** Proportion of Adults Reporting a Stroke Who Met the 2018 Physical Activity Guidelines for Americans, Stroke Belt States vs Non–Stroke Belt States, Behavioral Risk Factor Surveillance System, 2019^a^

Respondent characteristic	Total stroke respondents (n = 16,069)	Survivors in SBS (n = 3,555)	Survivors in NSBS (n = 12,514)	OR (95% CI)
		n (%)		
Aerobic guideline met^b^^,^ c	3,723	701 (19.7)	3,022 (24.1)	0.77 (0.69–0.86)
Strengthening guideline met^d^	2,137	509 (14.3)	1,628 (13.0)	1.10 (0.99–1.20)
Both guidelines met^b^	2,901	542 (15.2)	2,359 (18.9)	0.77 (0.70–0.86)
Neither guideline met^b^	7,308	1,803 (50.7)	5,505 (43.9)	1.31 (1.22–1.41)

Abbreviations: NSBS, Non–Stroke Belt states; OR, odds ratio; SBS, Stroke Belt states.

a SBS are Alabama, Arkansas, Georgia, Louisiana, Mississippi, North Carolina, South Carolina, and Tennessee.

b ORs assess differences between SBS and NSBS; χ^2^ *P* value <.001.

c Performing ≥150 minutes per week of moderate aerobic physical activity (10).

d Engaging in exercises to strengthen the muscles performed for 20 minutes at least 2 days a week (10).

Compared with stroke survivors in NSBS, stroke survivors in SBS reported greater difficulty walking or climbing stairs (OR = 1.33; 95% CI, 1.24–1.42; *P* < .001), dressing and/or bathing (OR = 1.26; 95% CI, 1.15–1.38; *P* < .001), and completing errands independently (OR = 1.24; 95% CI, 1.15–1.34; *P* < .001). Stroke survivors in SBS were more likely to report more than 2 weeks of unhealthy mental health (OR = 1.17; 95% CI, 1.08–1.27; *P* < .001) and more than 2 weeks of unhealthy physical health (OR = 1.22; 95% CI, 1.14–1.31; *P* < .001) per month compared with those in NSBS (Table 5).

**Table 5:** Physical and Mental Health-Related Quality of Life Among Stroke Respondents in Stroke Belt and Non–Stroke Belt States, Behavioral Risk Factor Surveillance System, 2019^a^^,^ b

Respondent characteristic	Total stroke respondents (n = 18,745)	Survivors in SBS (n = 4,272)	Survivors in NSBS (n = 14,473)	OR (95% CI)^c^
	n (%)			
**Do you have difficulty with...**				
Walking or climbing stairs?				
Yes	9,019 (49.8)	2,268 (55.3)	6,751 (48.3)	1.33 (1.24–1.42)
No	9,075 (50.2)	1,836 (44.7)	7,239 (51.7)	
Dressing and/or bathing?				
Yes	2,914 (16.1)	764 (18.6)	2,150 (15.4)	1.26 (1.15–1.38)
No	15,191 (83.9)	3,348 (81.4)	11,843 (84.6)	
Completing errands alone?				
Yes	4,427 (24.2)	1,180 (28.9)	3,427 (24.6)	1.24 (1.15–1.34)
No	13,903 (75.8)	2,910 (71.1)	10,476 (75.4)	
**Health-related quality of life**				
Mental score^d^				
<14	14,326 (79.2)	3,159 (77.1)	11,167 (79.8)	1.17 (1.08–1.27)
≥14	3,768 (20.8)	938 (22.9)	2,830 (20.2)	
Physical score^d^				
<14	11,359 (63.6)	2,418 (60.0)	8,941 (64.6)	1.22 (1.14–1.31)
≥14	6,510 (36.4)	1,615 (40.0)	4,895 (35.4)	

Abbreviations: NSBS, Non–Stroke Belt states; OR, odds ratio; SBS, Stroke Belt states.

a SBS are Alabama, Arkansas, Georgia, Louisiana, Mississippi, North Carolina, South Carolina, and Tennessee.

b Values for n may not sum to total because of nonresponse.

c ORs assess differences between SBS and NSBS; χ^2^ *P* value <.001 for all.

d Mental and physical scores are the number of “unhealthy days” reported in the last month.

## Discussion

Across all variables examined, the odds of having a stroke were significantly higher among those living in the 8 SBS compared with the NSBS, which is similar to what Howard et al found using the 2016 BRFSS (5). 

### Demographics and social determinants of health

Previous stroke prevalence studies have traditionally focused on adults aged 65 years or older, because this age group has generally had higher stroke rates (5–7,12,13) as well as age- and stroke-associated risk factors such as hypertension, poor diet, lack of adequate physical activity, increased stress, and tobacco and substance abuse (1,14,15). Early in the 2000s, stroke trends among younger adults in SBS began rising at the same rate as that among older adults (16,17). Our findings confirm this trend and demonstrate that younger adults in SBS are at 1.20 times the odds of having a stroke compared with age-matched adults in NSBS.

In addition, increased stroke prevalence for both men and women is notable in SBS compared with NSBS. Similarly, major ethnic groups in SBS also demonstrate higher rates of stroke compared with their NSBS counterparts. However, Hispanic or Latino individuals had lower odds of having a stroke, which may be due to under-reporting, decreased access to care, or lower survey participation among this demographic group (18). Furthermore, individuals of Hispanic origin are less likely to be contacted if they chose to answer the survey in Spanish (18).

Although low income is a barrier to healthy living, educational attainment has been shown to be an appropriate surrogate measure for socioeconomic status and is associated with the risk of sustaining a stroke in a SBS (19). The BRFSS yielded similar odds for having a stroke in SBS among respondents with incomes <$20,000 and higher than $75,000; however, a steady decline in risk was seen as educational attainment increased. Thus, enhancing education on the risk factors for stroke, especially in regions with lower rates of educational attainment and among those living in a more rural setting, may result in more effective campaigns against stroke (20).

### Other modifiable cardiovascular risk factors

Risk factors such as BMI, hypertension, diabetes, and smoking have been connected to higher odds of stroke since as early as the 1980s (14,15). Despite this knowledge, Americans continue to demonstrate higher levels of risk across all these modifiable risk factors, which account for 26% to 53% higher odds of having a stroke in SBS than in NSBS. Physical inactivity remains an important, independent risk factor for stroke, yet 50.6% of respondents in SBS reported that they had not met either of the 2018 Physical Activity Guidelines for Americans, which increased the odds of a stroke among those in the SBS by 31% compared with those in NSBS (10,21,22). Such risk factors must be addressed in the context of the social determinants of health such as educational opportunities; housing; racism and cultural sensitivity, which affect stress levels; and policies and environmental supports that promote food security, healthy eating, and active living across the lifecycle (23).

### Physical activity, disability, and health-related quality of life

While demographics and determinants of cardiovascular health are important in understanding stroke risk, a person’s perception of their health and functional ability can affect their function and participation in everyday activities. Perception of reduced physical or mental health could lead to further decreases in functional independence, as well as an increased risk for subsequent stroke and increased cost for the patient and society (24–26). This vicious cycle begins once a stroke survivor returns home from their rehabilitation and undergoes a subsequent deterioration of health and increased disability due to lack of physical activity and socialization, and decreased feelings of self-worth (24–26). Our findings support this cycle, as stroke survivors in SBS had higher odds of self-reported mental and physical unhealthy days than survivors in NSBS. The HRQOL metric was originally developed in 1993 and has been a constant BRFSS feature since 2000 (11). It is closely correlated with the domains of physical activity, obesity, smoking and substance abuse, mental health, violence and injury, environmental quality, and access to health care (11,24–26). For example, a person living with a chronic stroke with poorer mental and physical health may be less likely to be active in their community, may avoid social situations, and self-limit their participation in healthy activities due to negative beliefs (24–26). Increased likelihood of difficulty with walking or climbing stairs, personal hygiene, or completing tasks independently, which our findings show, may reinforce feelings of inadequacy and doubt and reduce positive health behaviors (24–27).

### Limitations

Our study has limitations related to BRFSS data. The surveys are cross-sectional and rely on self-report via landline and mobile telephone surveys, so findings cannot be determined to be causative. However, significant associations can generate hypotheses regarding the potential direction of these associations. Although the survey sampling is stratified and systematically collected, there is a possibility that individuals or groups may be underrepresented through random-digit-dialing, or because the respondent refused to participate. To mitigate this limitation, the sample generates state-specific examples, which when aggregated provide a large, nationwide population available for analysis. Issues of seasonality are addressed since the survey data are collected monthly, with a current sample size of more than 400,000 adults aged 18 years or older. Furthermore, in 2011, CDC increased the scope of its telephone calls to include more cellular telephones, as 3 in 10 individuals are estimated to only use cellular telephones rather than landline house phones, with this number increasing every year (8).

Due to the cross-sectional nature of the BRFSS, we cannot provide information on the causes and effects of stroke. However, our analyses confirm that the prevalence of stroke increases in association with several clinical, behavioral, and social variables among stroke survivors living in SBS. Furthermore, these findings provide evidence for a specific geographic Stroke Belt that contributes to increased illness and disability from stroke compared with other regions in the US and, therefore, should take precedence in providing tailored policies, clinical practices, and community programs directed toward addressing the health disparities.

### Suggestions for approaches to reduce disparity in the Stroke Belt

Numerous potential approaches for addressing these health disparities in the US provide some evidence of success. The FAST initiative, originally adopted from the United Kingdom, began in the US in 1999 as a 3-item Cincinnati Prehospital Stroke Scale which quickly assessed a person’s face, arm, and speech (FAS) (28). By 2007, multiple scales existed and, over time, FAST was able to identify almost 89% of individuals with ischemic strokes or transient ischemic attacks, thus capturing a large proportion of people with stroke (28). BE-FAST further expanded the mnemonic to capture often overlooked signs such as sudden balance or vision impairments (29). With increased awareness of stroke signs and symptoms, programs were designed for primary prevention in at-risk populations as stroke trends began to rise again.

Two examples of niche programs targeting stroke prevention strategies are “Live to the Beat” and “Start Small, Live Big.” “Live to the Beat” is a US campaign among Black adults that encourages heart-healthy lifestyles by creating a community of information and a culture of mindfulness, access to medical information, and easy-to-understand and easy-to-follow strategies to incorporate into everyday life (30). “Start Small, Live Big,” also called “Heart Health Steps,” is directed toward those aged 55 years or older to initiate or continue physical activity, eat healthy foods, and access medical care and information (31). These programs are only examples of a large number of programs that have seen some success by creating a community of people striving toward a goal, increasing awareness in historically underrepresented populations, and demonstrating that health is a life-long journey and you can start where you are.

Our findings call for further attention and innovation across the nation but especially among SBS to increase health education on stroke risk, including primary and secondary prevention, and rehabilitation. Emphasizing the benefits of physical activity and exercise, independent living skills, adequate access to nutrition and medical care, and socialization are important for primary (ie, having the initial stroke) and secondary (ie, subsequent strokes and disability from them) prevention. Health care professionals need to incorporate education, identify and mitigate individual risk factors, and contribute to the establishment of regular physical activity programming within their poststroke rehabilitation plans of care. Our findings also demonstrate a biphasic age distribution of stroke prevalence with an increasing trend in those aged 18 to 64 years (3) and the likelihood of stroke doubling every decade starting in the sixth decade (1). Thus, making primary prevention programs is a needed priority to reach people well before stroke risk develops.

Using these data, policy makers and public health campaigns should seek to create a culture embracing lifespan-specific healthy living, including regular physical activity and engaging in play at a young age, socialization and sport or regular physical activity in early adulthood, family-centric opportunities and plans for those with children, adherence to physical activity through middle and late life stages, and addressing health disparities and consistent access to healthy food across geographic regions (32,33). A multi-modal focused approach is needed to increase education and investing in infrastructure that influences physical activity (eg, active transport, public transportation, bicycle and pedestrian infrastructure, parks) and access to healthier food options and education on food that fits into a person’s cultural experience. Moreover, policies should be revised to allow for rehabilitation throughout an extended continuum of stroke recovery. The average poststroke length of stay ranges from 8 to 22 days in inpatient rehabilitation, with minimal follow-up or maintenance support (34). Reframing and prioritizing resources for extended stroke rehabilitation is imperative to break the vicious cycle of negative health perception and build positive health habits that can mitigate disability and related rising health care costs.

### Conclusion

Stroke is a neurologic and cardiovascular condition affecting a large proportion of people in the US every year, especially in SBS. While deaths and DALYs trended downward in the 2010s, both nationally and in SBS, that trend has reversed. As our findings indicate, living in SBS increased the odds of experiencing and living with the sequelae of stroke for all variables analyzed in this study. Furthermore, individuals in SBS show increased odds of experiencing more than 14 mentally and physically unhealthy days in a month, along with decreased functional independence and reduced likelihood of regular physical activity and exercise. The rising stroke prevalence in SBS demands specific primary prevention strategies implemented throughout the lifespan to decrease the likelihood of experiencing a stroke. State and local public health professionals and officials can use our findings to prioritize funding to create reasonable educational initiatives and feasible stroke prevention measures to improve the health status of their respective state residents.
